# Communications Just Received

**Published:** 1876-03

**Authors:** 


*Communications just Received.*—Hooping Cough, by Dr. T. I. Heard, Galveston, Texas; Chloride of Soda in Cancerous Ulcers, by Dr. G. M. Rivers, Walterborough, S. C.; A Communication, Dr. John Costen, Bowden, Ga.; Odservations in Practice, by Dr. W. L. Lipscomb, of Columbus, Miss.; the Opium Habit, by Dr. A. A. Lyon, Shreveport, La.; Cases in Practice, by A. R. Kilpatrick, Navasoto, Texas; Loss of Crystalline Lens by blow on Eyeball, by Thomas H. Kenan, Second Assistant Pysician, Georgia Lunatic Asylum.

